# *TET1* mutations as a predictive biomarker for immune checkpoint inhibitors in colon adenocarcinoma

**DOI:** 10.1186/s12957-022-02581-7

**Published:** 2022-04-08

**Authors:** Tianzhu Qiu, Xiaoxuan Wang, Furong Du, Xiangjing Hu, Fujun Sun, Chao Song, Jie Zhao

**Affiliations:** 1 Department of Oncology, The First Affiliated Hospital of Nanjing Medical University, Nanjing, 210029 Jiangsu China; 2 State Key Laboratory of Translational Medicine and Innovative Drug Development, Jiangsu Simcere Diagnostics Co., Ltd., Nanjing, 210042 Jiangsu China; 3 Henan Key Laboratory of Precision Medicine, Zhengzhou, 450052 Henan China; 4 National Engineering Laboratory for Internet Medical Systems and Applications, The First Affiliated Hospital of Zhengzhou University, Zhengzhou, 450052 Henan China

**Keywords:** Colon adenocarcinoma, Ten-eleven translocation 1, Immune checkpoint inhibitors, Overall survival, Predictive biomarker

## Abstract

**Background:**

The ten-eleven translocation 1 (TET1), which is essential for active DNA demethylation, plays a multifaceted role in the pathogenesis of colorectal cancer. The study has demonstrated the association of *TET1* mutations with a high response to immune checkpoint inhibitors (ICIs) in diverse cancers. However, the relationship between *TET1* mutations and the response to ICIs in colon cancer is still lacking.

**Methods:**

The prognosis, predictive markers, immune characteristics, mutation number of DNA damage repair (DDR) pathways, pathway enrichment, and drug sensitivity conditions were all compared between *TET1*-mutated and wild-type patients with colon adenocarcinoma (COAD).

**Results:**

The overall survival of patients with *TET1* mutations in the ICI-treated cohort was significantly longer than those without (*p* = 0.0059). Compared with the wild-type patients, *TET1*-mutated patients had higher tumor mutational burden and neoantigen load, enhanced abundance of tumor-infiltrating immune cells, increased expression of immune-related genes, and mutation number of DDR pathways. Additionally, the patients with *TET1* mutations were found to be more sensitive to lapatinib and 5-fluorouracil.

**Conclusion:**

These findings suggest that *TET1* mutations may serve as a potential biomarker for the response to ICIs in COAD patients.

**Supplementary Information:**

The online version contains supplementary material available at 10.1186/s12957-022-02581-7.

## Background

Colorectal cancer (CRC) is the third most commonly diagnosed malignant tumor and the fourth most deadly cancer worldwide, with nearly 0.9 million deaths each year [1, 2]. Its burden is projected to increase by 60% to over 2.2 million new cases and 1.1 million deaths by 2030 [3]. Compared with rectal cancer, colon cancer is more frequent, particularly in industrialized countries [4]. Each year, approximately 0.25 million new cases of colon cancer are diagnosed in Europe, representing around 9% of all the malignancies [4]. Despite significant advances in early detection and surgery, the 5-year survival rate of patients with metastatic CRC remains low, maintaining at around 14% [5].

In recent years, immunotherapy, a therapeutic schedule that makes use of the body’s own immune system to attack cancer, has shown promise for certain cancers, such as lung cancer and melanoma [6–8]. In a specific subset of CRC with mismatch-repair deficiency (dMMR) and microsatellite instability-high (MSI-H), immune checkpoint inhibitors (ICIs) are also found effective [9]. Unfortunately, ICIs do not respond well in CRC patients with mismatch-repair proficiency (pMMR) and microsatellite instability-low (MSI-L) [9]. Increasing evidence suggests the association of DNA methylation with immunotherapy/anti-immunity [10, 11]. The aberration of DNA methylation is considered a determinant of the tumor response to host immune activity, and its loss can facilitate immune evasion of tumors with high mutation and copy number load [12]. In a multicenter, retrospective study, DNA methylation signature was identified to be correlated with improved progression-free survival and overall survival (OS) in patients with stage IV non-small cell lung cancer (NSCLC) treated with anti-programmed death-1 (PD-1) agents [13]. Nevertheless, there are few studies reporting the correlation between genomic aberrations of DNA methylation-associated genes and response to ICIs.

As a member of the ten-eleven translocation (TET) family of oxygenases, TET1 can oxidize 5-methylcytosine (5-mC) to 5-hydroxymethylcytosine (5-hmC), which is essential for active DNA demethylation [14]. It plays a multifaceted role in the pathogenesis of CRC and can inhibit the proliferation of colon cancer by damaging β-catenin signal pathway [15, 16]. A previous study has demonstrated the association of *TET1* mutations with high responses to ICIs in NSCLC, bladder cancer, head and neck squamous cell carcinoma (HNSCC), melanoma, and esophagogastric cancer [17]. However, the relationship between *TET1* mutations and the response to ICIs in colon cancer is lacking.

In this study, we comprehensively characterized the landscape of *TET1* mutations in ICI-treated and non-ICI-treated colon adenocarcinoma (COAD) cohorts and then compared the copy number variants (CNVs) and prognosis between *TET1*-mutated and wild-type patients. Additionally, the tumor immunogenicity and antitumor immunity were also analyzed between *TET1*-mutated and wild-type patients.

## Methods

### Data source

In this study, 85 COAD patients treated by anti-PD-(L)1 monotherapy or anti-cytotoxic T-cell lymphocyte-4 (CTLA-4) combined with anti-PD-(L)1 therapy were selected from Memorial Sloan Kettering Cancer Center (MSKCC) and were considered as the ICI-treated cohort. The detailed information of these patients, such as clinical annotation, mutation data, and response data, was accessed from the cBioPortal for Cancer Genomics (http://www.cbioportal.org) [18]. As described by Hira Rizvi, the information on somatic mutations from MSKCC database was analyzed using targeted next-generation sequencing (NGS) [19].

Another 315 COAD patients not treated with any ICIs were from The Cancer Genome Atlas (TCGA), serving as the non-ICI-treated cohort. The data of gene expression (Illumina HiSeq, RNA-Seq), somatic mutations, OS, and disease-free survival (DFS) from the cBioPortal were analyzed. In addition, the Genomics of Drug Sensitivity in Cancer (GDSC) dataset used in this study was obtained from GDSC database (https://www.cancerrxgene.org) comprising the data of somatic mutations and drug sensitivity on COAD cell lines [20].

### CNV analysis

The Affymetrix single-nucleotide polymorphism (SNP) 6.0 microarray data of the non-ICI-treated cohort were accessed from the cBioPortal. The CNV segments with GISTIC 2.0 were analyzed using GenePattern (https://cloud.genepattern.org/gp/pages/index.jsf) [21]. Default values were endowed for all the CISTIC 2.0 variables.

### Prognostic assessment

According to the status of *TET1* mutations, COAD patients in both cohorts were divided into *TET1*-mutated and wild-type groups. In the ICI-treated cohort, the OS between two groups was evaluated using Kaplan-Meier method and compared by the logrank test. In the non-ICI-treated cohort, both OS and DFS were assessed. The tumor mutational burden (TMB) in the non-ICI-treated cohort was calculated by utilizing nonsynonymous mutations as the raw mutation count and dividing by 38 Mb [22], but in the ICI-treated cohort, the TMB score was used.

### Analysis of tumor immunogenicity and antitumor immunity

In the non-ICI-treated cohort, the abundance of tumor-infiltrating immune cells was estimated using tumor immune estimation resource (TIMER) algorithm [23]. Wilcoxon test was employed to compare the expression levels of genes related to cytolytic activity, chemokines, immune checkpoints, immune cells, antibody presentation, and antitumor immunity between *TET1*-mutated and wild-type groups. In this study, cytolytic activity was defined as the log average (geometric mean) of granzyme A and perforin 1 expression in transcripts per million [24]. The antitumor immune response could be reflected through the cancer immunity cycle, and the activities of the primary steps in this cancer immunity cycle were evaluated using a single sample gene set enrichment analysis (GSEA) according to the gene expression of individual samples [25].

### DNA damage repair (DDR) pathway mutations and pathway enrichment

The Molecular Signatures Database (MSigDB) of the Broad Institute comprised the gene sets related to DDR pathways. In both ICI-treated and non-ICI-treated cohorts, the number of nonsynonymous mutations in DDR pathway-associated genes was compared between *TET1*-mutated and wild-type groups.

In the non-ICI-treated cohort, the genes with differences in expression between *TET1*-mutated and wild-type groups were first identified through the edgeR package and then input into the clusterProfiler package in R for GSEA.

### Efficacy of targeted drugs and drug sensitivity analysis

Computational analysis of resistance (CARE), a computational method focusing on targeted therapies, was applied to assess the efficacy of targeted drugs. It could deduce the genome-wide transcriptomic signatures of drug efficacy based on cell line compound screens and identify genome-scale biomarkers for the response to targeted therapies [26]. The CARE score for each gene showed the correlation between molecular mutations and drug efficacy. Positive CARE scores represented the presence of mutations related to drug responses, while negative scores showed the presence of mutations associated with drug resistance.

The cell lines corresponding to the code of COAD in the GDCS database were assigned into two groups based on the status of *TET1* mutations, and then the cells in these two groups were compared for the sensitivity to molecular-targeted drugs and chemotherapy drugs.

### Statistical analysis

Enumeration data including gene mutations, gender, and drug types were compared using chi-square test or Fisher’s exact test, while measurement data like age, TMB, neoantigen load, abundance of immune cells, and immune-associated gene expression were compared by Mann-Whitney *U*-test. Clinical outcomes were assessed using Kaplan-Meier method and compared by the logrank test. All the statistical analyses used in this study were managed by R software (version 4.02, R Foundation, Vienna, Austria). The value of *p* ≤ 0.05 was considered statistically significant.

## Results

### Landscape of TET1 mutations in COAD

**Fig. 1:**
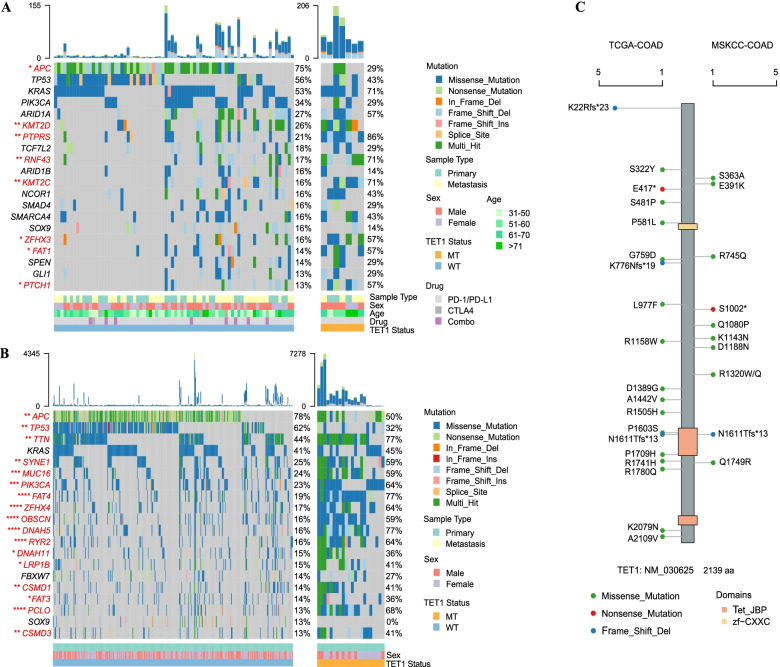
Landscape of *TET1* mutations in COAD. **A** The frequencies of mutant genes and clinical features between *TET1* wild-type and mutated patients in the ICI-treated cohort (MSKCC-COAD). **B** The frequencies of mutant genes and clinical features between *TET1* wild-type and mutated patients in the non-ICI-treated cohort (TCGA-COAD). **C** Comparison on the mutation sites of *TET1* gene in MSKCC- and TCGA-COAD cohorts

In the ICI-treated COAD cohort (*n* = 85), there were 7 patients harboring *TET1* mutations, with the mutational frequency of 8.2%. The common mutated genes in these 7 patients were listed in Table S1. Among *TET1* wild-type patients (*n* = 78), the top 20 mutant genes included *APC* (75%), *TP53* (56%), *KRAS* (53%), *PIK3CA* (34%), *ARID1A* (27%), *KMT2D* (26%), *PTPRS* (21%), and more, while in *TET1*-mutated patients (*n* = 7), the top 20 mutant genes comprised *KMT2D* (86%), *PTPRS* (86%), *KRAS* (71%), *KMT2C* (71%), *ARID1A* (57%), *ZFHX* (57%), *FAT1* (57%), *PTCH1* (57%), and more (Fig. 1a). By comparison to the frequencies of mutant genes and clinical features between *TET1* wild-type and mutated groups, the differences were pronounced in *APC*, *KMT2D*, *PTPRS*, *RNF43*, *KMT2C*, *ZFHX3*, *FAT1*, and *PTCH1* mutations.

In the non-ICI-treated COAD cohort (*n* = 315), *TET1* mutations occurred in 22 cases, with the mutational frequency of 7.0%. The frequent mutated genes in these 22 patients were described in Table S2. The frequencies of mutant genes and clinical features between *TET1-*mutated and wild-type groups were compared in Fig. 1b, where significant differences were presented in the majority of mutant genes including *APC*, *TP53*, *TTN*, *SYNE1*, *MUC16*, *PIKDCA*, and more. In addition, the mutation sites of *TET1* gene in the ICI-treated cohort and non-ICI-treated cohort were analyzed. It can be seen that N1611Tfs*13 missense mutations occurred in these two cohorts (Fig. 1c).

### CNV comparison

**Fig. 2:**
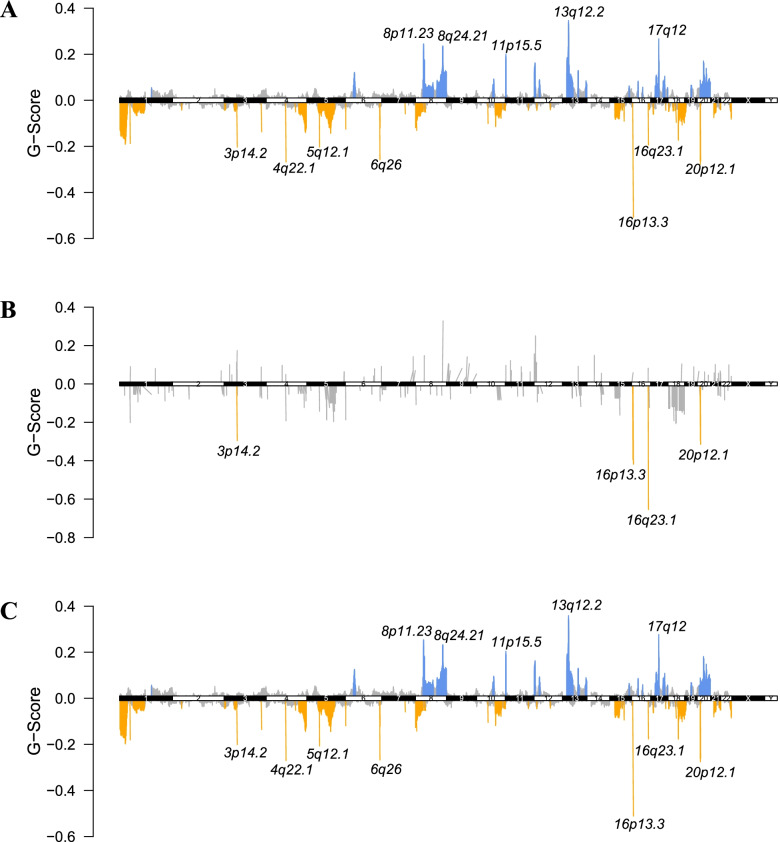
Comparison on copy number variants (CNVs) between *TET1-*mutated and wild-type patients in the non-ICI-treated cohort. **A** CNV status in COAD. **B** CNV status in *TET1-*mutated patients. **C** CNV status in *TET1* wild-type patients

By comparing the CNVs at the chromosome arm level between *TET1-*mutated and wild-type patients in the non-ICI-treated cohort, we found that *TET1* wild-type patients had distinct amplifications at 8p11.23, 8p24.21, 11p15.5, 13q12.2, and 17q12, as well as deletions at 4q22.1, 5q12.1, and 6q26 than those with *TET1* mutations (Fig. 2). The amplifications in *TET1*-mutated patients were indistinctive, and some deletions were more severe, especially at 16q23.1.

### Comparison of the prognosis and predictive biomarkers

**Fig. 3:**
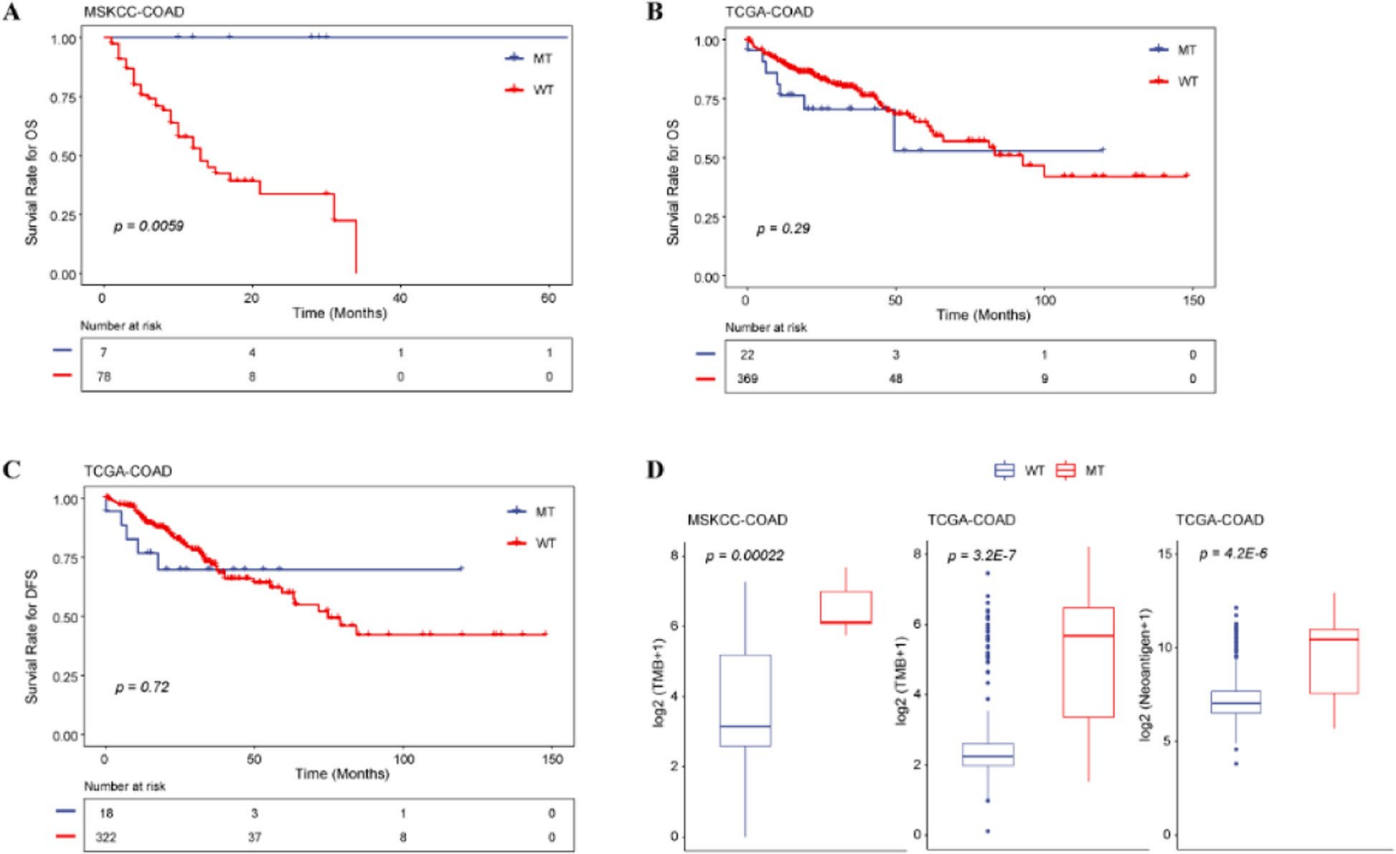
Comparison on the prognosis and predictive biomarkers between *TET1-*mutated and wild-type patients. **A** Analysis of the overall survival (OS) in the ICI-treated cohort (MSKCC-COAD), OS (**B**), and disease-free survival (**C**) in the non-ICI-treated cohort (TCGA-COAD). **D** Tumor mutational burden (TMB) levels in the ICI-treated cohort and TMB and neoantigen load in the non-ICI-treated cohort

In the ICI-treated cohort, the *TET1*-mutated patients had longer OS than the wild-type ones (*p* = 0.0059, Fig. 3a), while in the non-ICI-treated cohort, no significant differences were shown in both OS and DFS between *TET1-*mutated and wild-type patients (*p* = 0.290, Fig. 3b; *p* = 0.720, Fig. 3c). These findings suggested that COAD patients with *TET1* mutations might benefit from ICI therapy.

Considering that TMB and neoantigen load could serve as potential predictive biomarkers for the response to ICIs, the TMB and neoantigen load between *TET1-*mutated and wild-type patients were compared in this study. The results showed that the TMB and neoantigen load were significantly higher in *TET1*-mutated patients than in wild-type patients (Fig. 3d).

### Comparison of the tumor immunogenicity and antitumor immunity

**Fig. 4:**
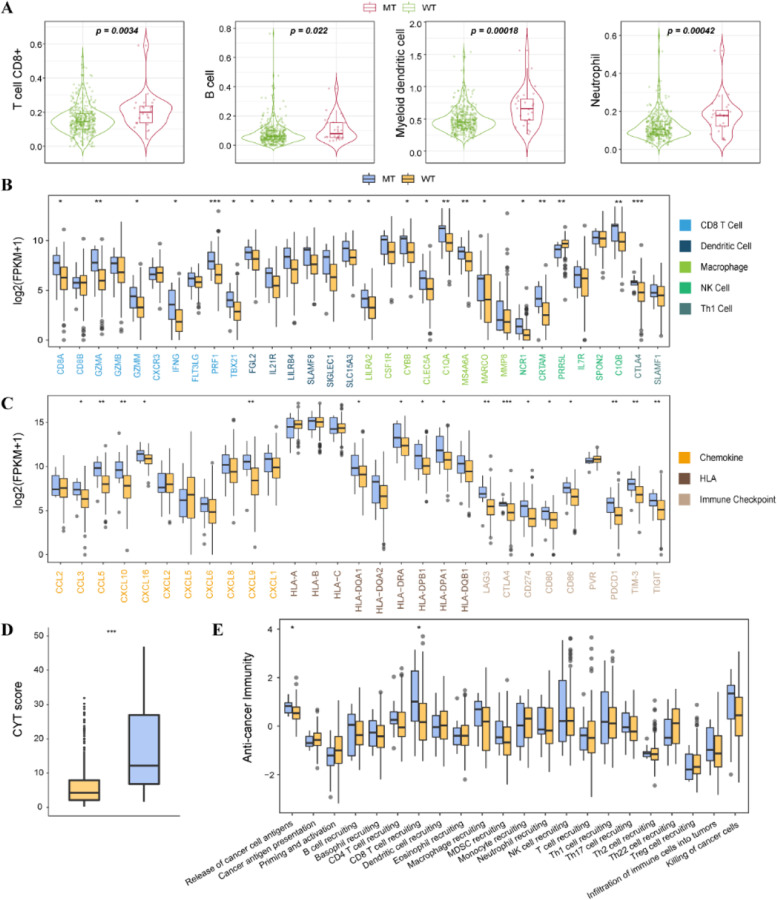
Comparison on tumor immunogenicity and antitumor immunity between *TET1-*mutated and wild-type patients in the non-ICI-treated cohort. **A** The abundance of tumor-infiltrating immune cells. **B** Immune cell markers. **C** Chemokines, immune checkpoints, and antigen presentation-related molecules. **D** Cytolytic activity. **E** The process of antitumor immune response

For identifying the immune features of *TET1*-mutated COAD, the expression levels of immune-related genes were compared between *TET1*-mutated and wild-type patients. By analyzing the abundance of tumor-infiltrating immune cells, we found that the CD8^+^ T cells, B cells, myeloid dendritic cells, and neutrophils were more abundant in *TET1*-mutated patients than in *TET1* wild-type patients (Fig. 4a). Compared with *TET1* wild-type patients, *TET1*-mutated patients had significantly increased expression of mRNA associated with immune cell markers (CD8^+^ T cells, *CD8A*, *GZMA*, *GZMM*, *IFNG*, *PRF1*, *TBX21*; natural killer cells, *CRTAM*, *NCR1*, *PRR5L*; Th1 cells, *CTLA4*, *SLAMF1*; dendritic cells, *FGL2*, *IL-21R*, *LILRB4*, *SIGLEC1*, *SLAMF8*, *SLC5A3*; macrophages, *C1QA*, *CLEC5A*, *CYBB*, *LILRA2*, *MARCO*, *MS4A6A*; Fig. 4b), chemokines (*CCL3*, *CXCL10*, *CXCL16*, *CXCL9*, and *CCL5*; Fig. 4c), immune checkpoints (*CD274*, *CD80*, *CD86*, *TIM3*, *CTLA4*, *LAG3*, *PDCD1*, and *TIGIT*; Fig. 4c), antigen presentation-related molecules (HLA-DRA, *HLA-DRA1*, *HLA-DPB1*, *HLA-DQA1*; Fig. 4c), and cytolytic activity (Fig. 4d) [27, 28]. In addition, regarding the antitumor immunity, *TET1*-mutated patients also showed higher expression levels in the release of cancer cell antigens and CD8^+^ T-cell recruiting than wild-type patients (Fig. 4e).

### Comparison of the number of DDR pathway mutations

By contrast to *TET1* wild-type patients, *TET1*-mutated patients in the ICI-treated cohort were found to have a significantly increased mutation number of DDR pathways, including nucleotide excision repair (NER), Fanconi anemia (FA), homologous recombination (HR), and nonhomologous end joining (NHEJ). In the non-ICI-treated cohort, the mutation number of six DDR pathways in *TET1*-mutated patients was significantly more than that in *TET1* wild-type patients, such as base excision repair (BER), NER, mismatch repair (MMR), FA, HR, and NHEJ. In both cohorts, the total number of DDR pathway mutations was markedly higher in *TET1*-mutated patients than in wild-type ones (Fig. S1).

### GSEA of TET1 mutations in COAD

**Fig. 5:**
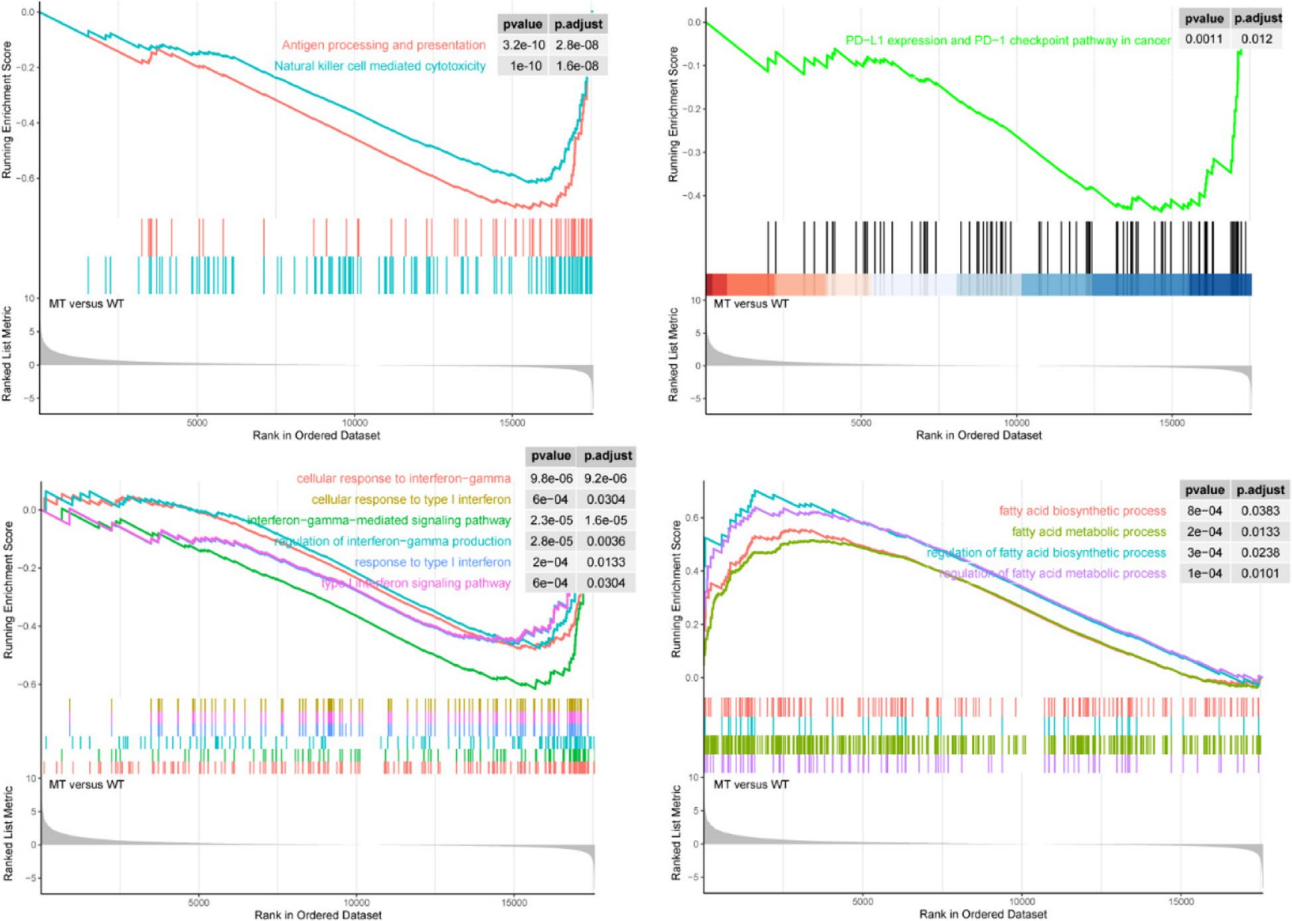
Pathway enrichment analysis of TET1 mutations in COAD patients

The non-ICI-treated cohort was used to investigate whether *TET1* mutations were involved in immune-related pathways. GSEA results revealed that the upregulation of fatty acid biosynthetic process and metabolic process was significantly enriched in *TET1*-mutated patients, while the downregulation of antigen processing and presentation, natural killer cell-mediated cytotoxicity, PD-L1 expression and PD-1 checkpoint pathway, interferon-gamma-mediated signaling pathway, interferon-gamma production, response to I interferon, and type I interferon signaling pathway was primarily enriched in *TET1* wild-type patients (Fig. 5).

### Association of TET1 mutations with drug responses and drug selection of COAD cells

**Fig. 6:**
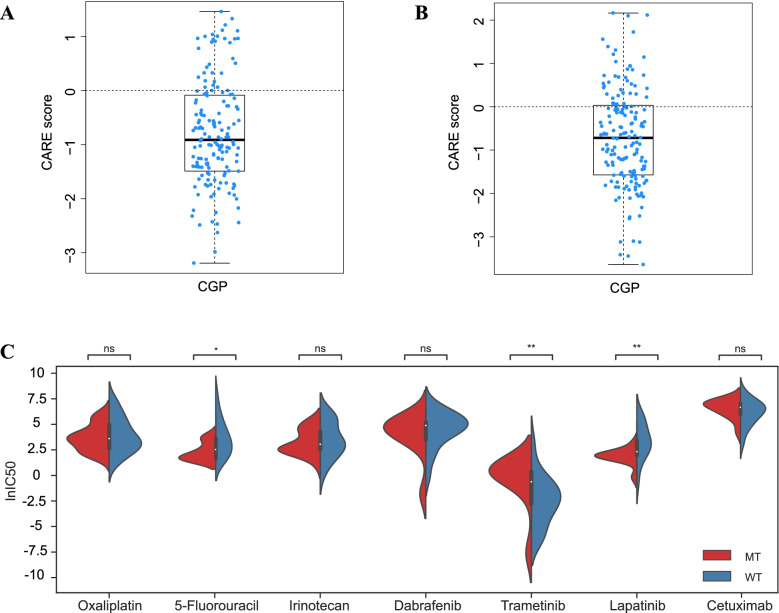
Association of *TET1* mutations with drug responses and drug selection of COAD cells. Correlations between *TET1* mutations (**A**), missense mutations (**B**), and drug responses (**C**). Violin plots of drug selection of COAD cells

In this study, the cell line data of antitumor drugs from Cancer Genome Project (CGP) were used to assess the association of *TET1* mutations with drug responses. The results showed that *TET1* mutations in COAD were associated with the drug resistance (Fig. 6a and b). Through analysis of GDSC dataset, *TET1*-mutated patients were found to be more sensitive to the molecular-targeted drug lapatinib and chemotherapy drug 5-fluorouracil, while oxaliplatin, irinotecan, cetuximab, dabrafenib, and trametinib were relatively resistant in *TET1*-mutated patients (Fig. 6c). These findings may provide some evidence for the drug selection in COAD patients with *TET1* mutations.

## Discussion

In the present study, MSKCC, TCGA, and GDSC datasets were used to investigate the effect of *TET1* mutations on the response to ICIs in COAD patients. The results revealed that *TET1*-mutated patients had better OS, higher TMB and neoantigen load, reinforced tumor immunogenicity, activated antitumor immunity, and more mutation number of DDR pathways than those without. In addition, the patients with *TET1* mutations were characterized by upregulated fatty acid biosynthetic process and metabolic process and more sensitive to lapatinib and 5-fluorouracil. These findings suggested that *TET1* mutations might be a potent predictive biomarker of immune responses and contribute to the enhancement of immune responses in COAD patients.

Recently, epigenetic characteristics have emerged as a leading feature of cancers. The gene expression is under the influence of epigenetic modifications, and abnormal epigenetic modifications play important roles in tumorigenesis and progression, especially DNA cytosine hydroxymethylation [29, 30]. TET enzymes can result in DNA demethylation and gene regulation by catalyzing the oxidation of 5-mC to 5-hmC [31]. The loss of 5-hmC related to *TET* downregulation and/or its functional changes is considered as a hallmark of cancer [32]. In multiple cancer types like colon cancer, this dysregulation has been identified [16]. There is evidence showing that TET proteins are involved in shaping the course of immune responses [33]. Of 21 DNA methylation-associated genes detected, mutations in *TET1* were observed to be significantly associated with better responses to ICIs in patients with NSCLC, bladder cancer, HNSCC, melanoma, and esophagogastric cancer [17], but its potential role in colon cancer was not analyzed. This study first assessed the relationship between *TET1* mutations and responses to ICIs in COAD patients. The results exhibited that the ICI-treated patients with *TET1* mutations had better OS than those without, while survival benefits were not significantly presented in the non-ICI-treated cohort, indicating that COAD patients with *TET1* mutations might benefit from ICI therapies.

The tumor immunogenicity is essential for the initiation of antitumor immune responses. High-frequency somatic mutations can improve the immune killing capabilities of T cells to tumor cells by inducing tumor cells to generate more neoantigens [34]. The tumor immunogenicity is influenced by several factors like antigen processing efficiency and presentation of dendritic cells in tumor microenvironment associated with the efficacy of ICIs in CRC patients [35, 36]. For instance, enhanced CD8+ cell response was correlated with ICI therapies [37]. Moreover, T-cell inflamed gene expression profile was found to be associated with the clinical benefits of patients treated with ICIs [38]. In the study made by Jiang et al., the tumor cytotoxic T-lymphocyte levels were evaluated via the average expression levels of CD8A, CD8B, GZMA, GZMB, and PRF1, and the results showed that the survival time of highly infiltrated cytotoxic T-lymphocyte patients receiving ICIs was prolonged distinctly. CD8+ T cells were also involved in the reinforcement of immune infiltration and antitumor immunity through chemokines including CXCL9 and CXCL10 [39]. Hence, higher TMB and neoantigen load, more abundant CD8^+^ T cells, B cells, myeloid dendritic cells and neutrophils, and increased expression of mRNA related to cytolytic activity, chemokines, immune checkpoints, immune cell markers, and more might explain why ICIs were more effective in patients with *TET1* mutations than those without.

DDR pathways play crucial roles in maintaining genomic integrity. Their aberrations can promote genomic instability and are related to high TMB. Studies have demonstrated that the mutations in DDR pathways may be a potent biomarker for predicting the response to ICIs [40, 41]. In the present study, patients with *TET1* mutations in both cohorts were found to have a significantly increased mutation number of DDR pathways than those without. This might be a reason for high responses to ICIs in *TET1*-mutated patients. Additionally, our results also revealed the upregulation of fatty acid biosynthetic process and metabolic process in *TET1*-mutated patients. During tumor progression, fatty acids secreted into the microenvironment can affect the function of infiltrating immune cells and phenotypes with exacerbated cancer-stromal interactions [42]. Recent studies have shown the extraordinary importance of fatty acid cellular metabolism on T-cell differentiation [43]. In clinical settings, targeting metabolic pathways was identified to have a beneficial effect on the T-cell response [44].

One major superiority of the present study was that it first reported the correlation between *TET1* mutations and the response to ICIs in COAD patients. Nevertheless, some limitations still needed to be illustrated. First, this was a retrospective study with small sample size, which might affect the statistical power. Second, the effects of *TET1* mutations on the CNVs, tumor immunogenicity, antitumor immunity, GSEA, and drug sensitivity were not analyzed in the ICI-treated cohort due to use of a single-targeted NGS panel and the absence of gene expression data. Third, the patients included in the ICI-treated cohort lacked the information on the line of treatment and were treated with anti-PD-(L)1 monotherapy or CTLA-4 combined with anti-PD-(L)1 therapy; this treatment difference may influence the response to ICIs. In the future, more large-scale, prospective studies should be conducted to verify our results.

## Conclusions

The present study revealed that ICI-treated COAD patients with *TET1* mutations had better OS than those without, highlighting the importance of *TET1* mutations in COAD patients receiving ICIs. Moreover, in the context of *TET1* mutations, the tumor immunogenicity and antitumor immunity were reinforced. In short, *TET1* mutations may serve as a potential biomarker for the response to ICIs in COAD patients.

## Supplementary Information

**Additional file 1: Figure S1.** Comparison on the number of DDR pathway mutations between TET1-mutated and wild-type patients in the ICI-treated (MSKCC) and non-ICI-treated (TCGA) COAD cohorts. **Table S1.** The common mutated genes in 7 COAD patients harboring *TET1* mutations from the ICI-treated cohort (MSKCC). **Table S2.** The common mutated genes in 22 COAD patients with *TET1* mutations from the non-ICI-treated cohort (TCGA).

## Data Availability

The data that support the findings of this study are available from the first author and corresponding author upon reasonable request.

## Abbreviations

CRC: Colorectal cancer
dMMR: Mismatch-repair deficiency
MSI-H: Microsatellite instability-high
ICIs: Immune checkpoint inhibitors
pMMR: Mismatch-repair proficiency
MSI-L: Microsatellite instability-low
PD-1: Programmed death-1
NSCLC: Non-small cell lung cancer
TET: Ten-eleven translocation
5-mC: 5-methylcytosine
5-hmC: 5-hydroxymethylcytosine
HNSCC: Head and neck squamous cell carcinoma
COAD: Colon adenocarcinoma
CNVs: Copy number variants
CTLA-4: Cytotoxic T-cell lymphocyte-4
MSKCC: Memorial Sloan Kettering Cancer Center
NGS: Next-generation sequencing
TCGA: The Cancer Genome Atlas
OS: Overall survival
DFS: Disease-free survival
GDSC: Genomics of Drug Sensitivity in Cancer
TMB: Tumor mutational burden
TIMER: Tumor immune estimation resource
DDR: DNA damage repair
MSigDB: Molecular Signatures Database
GSEA: Gene set enrichment analysis
CARE: Computational analysis of resistance
NER: Nucleotide excision repair
FA: Fanconi anemia
HR: Homologous recombination
NHEJ: Nonhomologous end joining
BER: Base excision repair
MMR: Mismatch repair
