# Supplementary material for: TET1 mutations as a predictive biomarker for immune checkpoint inhibitors in colon adenocarcinoma
Source: World J Surg Oncol. 2022 Apr 8;20:115. doi: 10.1186/s12957-022-02581-7 (PMC8991851; doi:10.1186/s12957-022-02581-7)
Supplement: Supplementary file 1 — Additional file 1: Figure S1. Comparison on the number of DDR pathway mutations between TET1-mutated and wild-type patients in the ICI-treated (MSKCC) and non-ICI-treated (TCGA) COAD cohorts. Table S1. The common mutated genes in 7 COAD patients harboring TET1 mutations from the ICI-treated cohort (MSKCC). Table S2. The common mutated genes in 22 COAD patients with TET1 mutations from the non-ICI-treated cohort (TCGA). [file 12957_2022_2581_MOESM1_ESM.docx]

**Supplementary Materials**


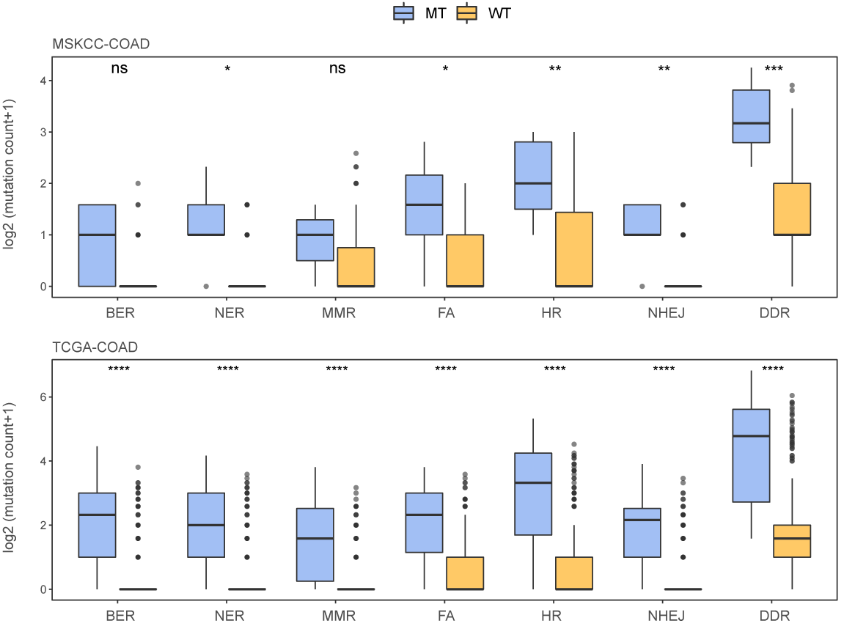


**Figure S1** Pathway enrichment analysis of *TET1* mutations in COAD patients.

**Table S1.** The common mutated genes in 7 COAD patients harboring *TET1* mutations from the ICI-treated cohort (MSKCC)

| Mutated genes | 1 | 2 | 3 | 4 | 5 | 6 | 7 |
| --- | --- | --- | --- | --- | --- | --- | --- |
| *TET1* | p.R745Q p.S1002* | p.N1611Tfs*13 p.S363A | p.E391K | p.D1188N | p.Q1080P p.K1143N | p.Q1749R | p.R1320W p.R1320Q |
| *KMT2D* | p.L4323S |  | p.L4231R | p.P2354Lfs*30 p.Q3919dup p.T698Hfs*6 | p.E551* | p.Q3974del | p.C5477Lfs*10 p.P727S |
| *PTPRS* | p.M1044I p.P164S p.T80M | p.A937V | p.P622Lfs*16 | p.R1492Q | p.F1406L |  | p.P622Lfs*16 |
| *B2M* |  | p.L15Ffs*41 p.T93Hfs*2 |  | p.V69Wfs*34 p.T93Lfs*10 | p.E64* p.Y87* | p.V69Wfs*34 p.S16Afs*27 | p.L59* |
| *CREBBP* | p.D2433G | p.I1084Sfs*15 |  | p.K1588del | p.L869F p.S71L |  | p.G2386R |
| *DOT1L* | p.R794K | p.G594D | p.P830L | p.A370T |  | p.P1312T |  |
| *KMT2C* | p.R4478* p.E718* | p.K1201Nfs*4 |  |  | p.S1576Y p.K1162Q p.R35I | p.R2463C p.P1537S p.A1361V | p.D1670V |
| *KRAS* | p.A146T p.T144P |  | p.Q61H | p.G13D | p.A146T | p.Q61K |  |
| *NOTCH3* |  | p.R1669H | p.C959Y | p.V523A |  | p.R1998C | p.G2090A |
| *RICTOR* |  |  | p.R508W | p.X195_splice | p.R1130Q | p.T268I | p.C959G |
| *RNF43* |  | p.G659Vfs*41 p.X125_splice | p.G659Vfs*41 | p.G659Vfs*41 p.R371* p.P229L |  | p.G659Vfs*41 p.P594del | p.G659Vfs*41 |
| *ATRX* | p.L11F |  |  | p.D1222N p.S1110del | p.E1113D p.N636T |  | p.K358Nfs*2 |
| *BCOR* | p.F977L | p.K100Rfs*85 | p.K1061Sfs*52 |  | p.N820K p.R188W |  |  |
| *BRCA2* | p.E2129* p.K2673T |  | p.V2174G p.I605Yfs*9 |  | p.F883L p.E954D p.N1277K p.S1606Y p.R1677I p.D2005Y p.E2129* p.S2667I |  | p.N1287Ifs*6 |
| *FAT1* | p.K1978Q p.S1652F p.D1399N p.D340Y p.E87K | p.S3594N |  | p.V812A | p.E2684* p.D2073Y p.A1663T p.R1627Q p.F1301V |  |  |
| *FLT4* |  | p.P30Rfs*3 |  |  | p.F1332L p.D105N | p.P30Rfs*3 | p.L1014V |
| *NF1* | p.E2607K | p.K428E | p.Y2398C |  | p.E1583* p.L2639I |  |  |
| *NOTCH1* | p.D740N p.D1546N | p.R1762Q | p.D469E | p.V1229Lfs*216 p.T712Pfs*60 |  |  |  |
| *NOTCH4* | p.P652S | p.G1557S |  | p.R234W |  |  | p.A1189V |
| *PIK3CB* | p.R620* p.E147* |  |  |  | p.L639I | p.G840D | p.A19V |
| *PIK3R1* | p.K592N p.S709Y | p.A183T p.H697Y | p.N527D p.G588Vfs*7 |  | p.R557* |  |  |
| *POLE* | p.P286R |  |  |  | p.F1672L p.S459F p.D275G | p.L698Cfs*94 | p.G1086V |
| *RAD50* |  |  | p.T410Lfs*5 | p.D69N p.R254H | p.R1093* | p.K722Rfs*14 |  |
| *ROS1* | p.R2184I p.E1974* p.N1090K p.E738D p.Q562H p.E395D p.S370Y |  |  | p.S296F | p.R2184I p.D1496Y p.R380K |  | p.S866N |
| *SETD2* | p.R1598Q p.E517* p.E346D |  | p.T1270M | p.S2382Lfs*29 |  |  | p.G1419C |
|  |  |  |  |  |  |  |  |

**Table S2.** The common mutated genes in 22 COAD patients with *TET1* mutations from the non-ICI-treated cohort (TCGA)

| Mutated genes | 1 | 2 | 3 | 4 | 5 | 6 | 7 | 8 | 9 | 10 | 11 |
| --- | --- | --- | --- | --- | --- | --- | --- | --- | --- | --- | --- |
| *TET1* | p.K776Nfs*19 p.D1389G p.N1611Tfs*13 p.R1780Q | p.P1603S | p.K22Rfs*23 p.P1709H | c.*32A>G | p.S322Y p.A1442V | p.E417* | p.L287L | p.P581L | p.G759D | p.K22Rfs*23 | p.S481P |
| *FAT4* | p.A450V p.P3508H |  | p.G972G p.L3734Yfs*8 | p.S2873N | p.A454A p.A2421T p.Y3978C p.P4401L | p.S536S p.A1534T p.E1642K p.K2001R p.D3505Y p.K3723K | p.V779L p.E904D p.D1343N p.R1788H p.R1815C p.K2096T p.I2153N p.T2473I p.R2685Q p.L2688R p.E2926D p.I2971T p.S3550N |  | p.G195C p.N775N p.M4853T | p.A868V | p.R356H p.E4497D |
| *DNAH5* | p.F4277L |  | p.P3316Lfs*22 | p.R3756M p.P3316Lfs*22 p.Y387Y | p.R4071H p.V3196V p.H1303Y | p.G3644E p.D2207Y p.Y1706H p.S321S p.E54K | p.T2251T p.I1698T p.R1668L p.Q1546Q p.E1493E p.S1280S p.X1088_splice p.R224Q |  | p.I1855Sfs*16 | p.A3053V | p.L1467Wfs*3 |
| *TTN* | p.G32077G p.I20093I p.X18038_splice p.R16331* p.C2544Y |  | p.L32868L p.P29821S p.A29770V p.I23397L p.P21803S p.G16882G p.I16557V p.P16342S p.S14444R p.A12650Gfs*4 p.P11612P p.V11199V p.K11145N p.E9671K p.K1264N | p.P19922Lfs*10 p.V1964Wfs*14 p.G1855G | p.K28560Nfs*89 p.R27734H p.V22291I p.A21327V p.P21243P p.D20259Rfs*40 p.L17749L p.G17518G p.A13778V p.T9261T p.G6469V p.G5543G p.E2216A p.V1565V | p.Y31190C p.L28029I p.L26036L p.S21362P p.I20148T p.E19310* p.E16482D p.S16440Y p.M15523I p.A12581T p.F9034V p.G8727* p.D8407N p.E8248K p.F6304L p.S3911Y p.E3697D p.S2669S | p.L34079I p.R32062Q p.R32033I p.X31648_splice p.T30129I p.S28357L p.R28225R p.D28191Y p.R27702C p.R26958C p.E26879* p.N26110T p.R26061* p.E25586* p.S25221S p.R23722C p.T23378A p.V23162G p.R19381C p.S18069G p.I16804S p.S15078Y p.C13500C p.E11103* p.F11094V p.L11068L p.F8563L p.I7978M p.C7751C p.D7466G p.S7345Y p.A7299V p.L6670M p.L6504I p.K5649N p.E5575* p.Y5335Y p.V5331V p.V4743V p.D1340E p.Q1073H p.L326L |  | p.T25858T p.D23928G p.A15369V p.V11008Sfs*13 p.T2952I | p.A32206D p.T31572I p.T24702S p.A17023D p.Q2321R | p.R33242H p.R32934H p.R24719C p.T9845M |
| *SYNE1* | p.Q3362dup p.L2564W p.E428Rfs*3 |  | p.H8050N p.Q6699R p.K6197K p.P222L | p.S1134S | p.Q5965Q | p.R6170I p.D6125N p.D5255N p.L3521I p.F2276C p.K2257E p.E2187* p.S1195Y | p.S8017Y p.R7690Q p.A7165A p.E6446D p.A6206V p.V5548V p.E5157E p.S4403L p.N4247H p.A3774V p.A3299V p.G2675G p.F2563L p.T986T p.V756V p.E137* p.S111P p.L99L | p.R1805Q |  |  | p.R1309Q |
| *DST* | p.G1327D |  | p.Q3497H | c.4417-1668G>C | c.4771-3034G>A | c.4770+4394T>C | p.K2520K p.E1845D p.E1716* p.A1400S p.F1059C p.D779Y p.W381L p.S49S p.L81I |  | c.4770+2478G>A c.4416+4512C>T | c.211+8225T>A | c.4416+4308delA c.4416+4186C>T |
| *FUT9* | c.*7298dupA |  | p.H150Q | c.*5667delA |  | p.S247A | c.*984A>G c.*3326G>T c.*1216T>G c.*1965C>A c.*4142G>A c.*4606G>T c.*7521C>A c.*9164C>A c.*10341G>T |  | c.*2230delT | c.*2230delT c.*3423G>T | c.*5882G>T |
| *PCLO* |  |  | p.G2989E p.V1883Ffs*29 p.R1822M | p.E4529K | p.T4546M p.K1212K | p.L1656R p.D1263G | p.T3447T p.F2648C p.E1953D p.S1356G p.T254P |  |  | p.D2060G | p.K2442Sfs*2 |
| *DYNC1H1* | p.A1775A p.S2624S |  | p.S1157A p.X4072_splice |  | p.A1096T | p.R1357* | p.C2639C | p.T659T | p.K3112Hfs*3 | p.C4121C | p.V3453I |
| *LAMA1* | p.P2433L p.N2395T p.A74T |  | p.S2424S p.E245K | p.K1267K p.N496Tfs*27 | p.T2016T | p.L1962R | p.K2849N p.V2786V p.S2021S p.E1935* |  | p.S2281Y p.G755S | p.Q1283H | c.3507+3A>G |
| *LRP2* | p.R3551H |  | p.C3635C p.R3343C | p.Q360K | p.G4113D p.A560T p.G304R | p.R2225* p.R679I | p.F4225F p.C3068Y p.G2974G p.A2595D p.A1493A p.G1341V p.N1019H |  | c.*100delA |  | p.N1280N |
| *MUC16* | p.T11856I p.T11847Qfs*15 p.T5326T |  | p.N9430Tfs*13 p.K6604Rfs*26 p.S4382Y p.A1036V | p.A8151T | p.R12149H | p.D12715N p.D11236Y p.P10725S p.S10342Y p.I8765I p.E8447* p.F8400L p.V8153V p.V6983V p.S5056Y p.S3711Y p.A3023T p.E2017D p.S1157Y p.E1076* p.E1066D | p.Q13823R p.L13771M p.A12101T p.S12060S p.T11591A p.L11545M p.E11480E p.E10522K p.R10422Q p.P9868P p.T9655A p.S9342P p.L9189L p.D8977Y p.F8952L p.E8951* p.S8833N p.S8708Y p.R8606H p.S8175P p.F6653L p.S6150Y p.I4872N p.T3327A p.V3224A p.E2719* p.T1423T p.A1011T |  | p.V2005V | p.A14356A p.P12426H p.D7202N | p.S741N |
| *OBSCN* | p.R1764W p.G3685S |  |  |  | p.R3186W p.A3865T | p.R6749C | p.I1718M;p.A3213V;p.V4027M;p.S6162S;p.E6181K;;p.I6349I |  | p.T4483A | p.D4377V | p.A3553V |
| *PIK3CA* |  | p.H1047R | p.H1047R |  | p.R88Q |  | p.C585C p.R777M | p.E545K |  |  | p.H1047R |
| *RYR2* | p.I4094V p.R4469Tfs*18 | p.R882I | p.E1127K p.G1444G p.P2677P p.D4195G |  | p.K1097N p.R4790Q | p.G197G p.E1364* p.N1502N p.L2130L p.E3424* p.L3670M p.K3914K p.I4145I p.F4480L p.R4497H p.D4664Y | p.K603K p.K671N p.E839K p.Q1287H p.P1307P p.S1424P p.I1830T p.E1867* p.R2258C p.Q2654R p.I2770S p.F2962L p.R3615W p.F3789F p.Q3955H p.K4436T p.S4583Lfs*11 p.I4831I |  | p.R1760Q p.K4352E |  | p.A2213Lfs*22 |
| *ZFHX4* | p.E468E p.A3418T |  | p.D484A p.Q806P p.V1580I |  | p.H1565Tfs*5 | p.E237D p.E330* p.V778V p.L1961L p.P2487L | p.E442D p.G754E p.G1940G p.R2303W p.S2867Y p.I3222I |  | p.R274M |  | p.N249Tfs*30 |
| *ABCA13* |  | p.V1110I | p.I2579* |  | p.G2858R | p.W578* p.V1590A p.E2143* | p.C468C p.E815D p.E2676* p.R4043H | p.K3855Nfs*139 | p.L1231P p.N1764Tfs*18 |  |  |
| *CNTNAP5* | p.G65D p.G964S |  |  | c.*909C>T c.*929_*932delCATA | c.*929C>T | p.F326Y p.F811V p.C956C p.R1234* | p.X177_splice p.R931H |  |  | p.D730V | p.P552S |
| *DNAH7* | p.A1394V |  | p.L2192Qfs*11 |  | p.V3433M | p.E3042* p.A1504V p.F1071L p.V177A | p.I3693I p.K826N p.D649Y p.E299* |  | p.R3386H | p.P2310Tfs*50 |  |
| *FBN3* | p.R2330R p.D1765N p.R848C |  | p.G1826C |  | p.P1708Rfs*31 | p.K790T | p.E2675G p.G2155G p.S1418S |  |  | p.Q1587Q | p.C1223Afs*11 p.R781Q |
| *HERC2* | p.S2792S p.T2738T | p.S2637del p.K1446R | p.L3306* p.N1574N |  | p.Y1924Y | p.F3846L | p.R2554* p.G684C p.V264V |  | p.W3700C | p.N4304S p.T3514A p.E695V | p.N3793S |
| *KMT2D* | p.R1614W | p.F2444F | p.Q2004* |  | p.P2354Lfs*30 |  | p.D1343G |  | p.A3552Sfs*4 p.P3102Lfs*17 p.T2956T p.G2651G p.A531A | p.R5351L p.L4069V p.M3870V p.R1687H |  |
| *USH2A* |  |  | p.S5144S p.S3344P p.N1848T p.F1488V |  | p.A5048V p.D4007N | p.A3605D p.L2892I p.F2369L p.P1368H p.E1288* p.D591Y p.F225L | p.S4696Y p.C3307R p.L1402I p.E1359K p.T701A p.R240I p.A146S |  | p.G660G | p.W354* |  |
| *VPS13B* | p.T2359T |  | p.N1245K p.G1584G |  | p.R3223Q | p.F1430L | p.R146Q p.F2475V |  | p.Q2962R | p.K1345E |  |
| *RYR1* |  |  | p.P1054H p.A2864V | p.R2965H | p.R2140Q p.P4491S |  | p.G1855G p.E1944E p.S3116L p.V4583V |  | p.S1007S |  | p.L1599L |

| Mutated genes | 12 | 13 | 14 | 15 | 16 | 17 | 18 | 19 | 20 | 21 | 22 |
| --- | --- | --- | --- | --- | --- | --- | --- | --- | --- | --- | --- |
| *TET1* | c.*79C>T | p.K22Rfs*23 p.A105A | p.R1158W | p.T559T | p.K2079N | p.A2109V | p.R1505H | p.N1273N | p.R1741H | p.K22Rfs*23 | p.L977F |
| *FAT4* | p.A596V p.S2676Y p.R3342Q p.L3734Ffs*22 p.R3830C p.S4383R p.M4919I | p.R152H p.G179D p.L2385S p.P3539T | p.F1841L p.G2130G p.R2871I p.S3112A p.K3289Q | p.A807T | p.Q4906Rfs*14 |  | p.R122Q p.A339V p.I1651T p.D4560E | p.G1794* | p.R1806H |  | p.T4004T |
| *DNAH5* | p.A4125V p.E4026K p.G1985D | p.R3098Sfs*14 | p.R3743I p.E1330K p.R917I p.R224Q | p.R3079* | p.R293Dfs*12 |  | p.P3316Lfs*22 p.G1976Efs*78 |  | p.R2909C |  | p.T4483M |
| *TTN* | p.R34348* p.F33267F p.D32837G p.R30504C p.R28725I p.L26326L p.R25372* p.V25212G p.N24861H p.F24845L p.L23696I p.I22834I p.P19454P p.D17750N p.R17490I p.R15620* p.I15413M p.R14257* p.S13828A p.E13604* p.G13493* p.D13166N p.W12888* p.P10213S p.F9566F p.F7480F p.D7451N p.R6811C p.V5956A p.G4690G p.D4347N p.P3972S p.E3813D p.A3707V p.R2506Q p.R1003C p.R220Q | p.P22170S p.F18705Lfs*8 p.N3698D | p.R25535R p.E21669G p.X19422_splice p.E9478D p.K9025R p.K7196T p.F6849L p.A6742D p.L4938* | p.A12758T p.L4363S p.K2407Nfs*10 p.R77R | p.P32871P p.R32605C p.V12225I |  | p.A21259A p.S7359S p.E7307G p.A7105Cfs*8 p.P1082S |  | p.V22573Cfs*4 p.V22116A p.A17496S p.E16119D p.R14491H p.S6179S p.G5399C |  | p.K1706M |
| *SYNE1* | p.A7909V p.E7825* p.S7253L p.Q6261* p.V5548V p.L3521I p.R3399* p.V3147G p.I2756L p.V1337A p.K1226N p.R81H | p.Q6964Q p.F243S | p.G7638G p.E7463D p.E7305* p.E6305* p.L4415I p.R3070* p.K1899N | p.F1557L | p.G2437S | p.N2333Kfs*2 |  |  | p.R8738R p.Q7258E |  |  |
| *DST* | p.E5313D p.V4635V p.E3239* p.F3141F p.X2369_splice p.E1759K p.E124* p.R68W |  | p.F216V |  | c.4771-2790T>C |  | c.4416+4308dupA |  | p.G5203G p.T4428Nfs*23 |  | p.S3376S |
| *FUT9* | p.K229T p.E278D p.E354D | c.*1917dupG c.*2230delT c.*5135G>A | c.*106T>G c.*211T>G c.*4205T>G c.*5770A>G |  | c.*1924delA |  | c.*2230delT | c.*4830G>A | c.*1924delA c.*9725delC |  |  |
| *PCLO* | p.I4777M p.R3313W p.K2743T p.K1007T p.R882R | p.R3493C p.C1109C | p.R3435Q p.E137* | p.K1240Sfs*78 | p.K2442Sfs*2 p.R1756L p.P296T |  | p.K2442Sfs*2 p.K1219Nfs*2 | p.A5115S | p.I247Lfs*42 |  |  |
| *DYNC1H1* | p.N215S p.E1016D p.R1360Q p.I1530I | p.Y146Y p.E1708D | p.R2705I |  | p.R3191Q p.R4123Q |  |  |  | p.D87D; |  |  |
| *LAMA1* | p.H1780H | p.G1193R p.N496Tfs*14 | p.D2946G p.X2593_splice | p.N1940N |  |  |  |  | p.R59Q |  |  |
| *LRP2* | p.C4389* p.R4319* p.S4053Y p.F3847F p.I3202M p.R2175W p.R679I p.A57A | p.R3086C | p.Y1419* p.S830L p.T554A | p.R3305R | p.S714Hfs*51 |  | p.H32Y |  |  |  |  |
| *MUC16* | p.T11395N p.A11107T p.N8324H p.T7568A p.P6588H p.G6160G p.A6111E p.G5161V p.E4853E p.V4020A p.R3931I p.E1306E p.E337* p.P186H | p.S7713Lfs*5 p.T6444N p.W3181R p.T1636del | p.Y14088* p.S11173Y p.F10842C p.E8764K p.S8085Y p.S7745R p.T7572A p.S6238P p.F5187L p.A4482V p.F4193L p.T3112P p.S1083F p.G426* p.E388* | p.T6708T p.E3891D |  |  |  |  |  | p.T13441P |  |
| *OBSCN* | p.R1842H p.D3578N p.R3669R p.A3740V p.R4856H p.V6675I |  | p.E1766D p.R4213H | p.G1966D | p.A4716A p.A5454T |  | p.A1309T p.G1974G |  | p.G24D p.A1889V p.D2843D |  |  |
| *PIK3CA* | p.E81* p.R88Q p.H1048R |  | p.R88Q p.M732I p.H1047Q |  | p.Y1021C | p.E542K |  | p.E545K p.E970K | p.P609H | p.G1007R | p.R108H p.M1043I |
| *RYR2* | p.G7G p.E1971* p.R2127W p.L3725I p.E4137K p.F4660L | p.G715Wfs*7 | p.A266T p.K546T p.E1237* p.I3218I p.F3319V p.N4178N p.K4791N p.L4858I |  | p.R485W p.D602Y |  | p.H576Y |  | p.R296Q p.R1254M p.V3557V p.Y3923C |  |  |
| *ZFHX4* | p.D2342G p.L2865I | p.A562T | p.G202R p.V269L p.F1722V p.E2286D p.L2762L p.S2805L |  |  |  | p.K2850Nfs*55 p.R3019C | p.G754R p.G3313V | p.V453I p.I939V p.D3416D | p.G2928V |  |
| *ABCA13* | p.N1614N p.A2159V p.V3115I p.D3470N p.K3519N p.X4599_splice |  | p.E2350* p.E3596D p.D4739Y | p.Q1252R |  |  | p.R4286Q | p.R4282H | p.F2876L |  |  |
| *CNTNAP5* | p.M133I p.X625_splice p.R931C | p.R643W | p.S901L |  |  |  | p.F292V p.P381P |  | p.F1002Lfs*17 | c.*929C>T |  |
| *DNAH7* | p.D2941Y p.I2236M p.L2229F p.R628I | p.K2448K | p.R1866* p.E122* p.K119T | p.P1564Tfs*5 |  | p.Y1656Y |  |  | p.V827* p.L367R |  |  |
| *FBN3* | p.R2510H p.R2199W p.E2149K |  | p.R2199W | p.T2578Pfs*120 p.V616A |  | p.T1105M | p.E1448K |  | p.G1901C p.G1440Vfs*14 |  |  |
| *HERC2* | p.V4404V |  | p.I3457I p.L2860R |  |  |  | p.S181Vfs*85 |  | p.V2244V |  |  |
| *KMT2D* | p.N3077D | p.T4629Pfs*11 | p.R4420W p.F1776L | p.G5410W | p.A2133T p.P1940T |  |  |  | p.Q3264K |  |  |
| *USH2A* | p.L4053V p.A3447A p.D528N p.R205I | p.R4608Q | p.G5168G p.G4794G p.L2987L p.K1802Q p.F1364L | p.P5077H | p.R1245T |  |  |  | p.G3853R p.X723_splice |  | p.Q4003L |
| *VPS13B* | p.D226N p.I2821V | p.L58* | p.R3837C |  |  |  | p.R1462H |  | p.I1123I | p.K1407R |  |
| *RYR1* | p.R1043H p.E1175K p.E2081G | p.G3578G | p.E798G p.E1077D |  | p.E456K p.F2517L p.V4670I |  | p.N4141N | p.E1332K p.V2341I | p.D2713Tfs*33 p.Q3836* p.S4235S |  |  |
